# Cognitive Performance Deficits Are Associated with Clinically Significant Depression Symptoms in Older US Adults

**DOI:** 10.3390/ijerph20075290

**Published:** 2023-03-28

**Authors:** Orestis Delardas, Panagiotis Giannos

**Affiliations:** 1 Promotion of Emerging and Evaluative Research Society, London AL7 3XG, UK; orestis.delardas.20@ucl.ac.uk; 2 Department of Life Sciences, Faculty of Natural Sciences, Imperial College London, London SW7 2AZ, UK

**Keywords:** depression, cognitive function, aging, older adults, NHANES

## Abstract

Accumulating research has described cognitive impairment in adults with depression, however, few studies have focused on this relationship during older adulthood. Our cross-sectional study investigated the association between cognitive function performance and clinically significant depression symptoms in older adults. We analysed the data from the 2011 to 2014 National Health and Nutrition Examination Survey on older (aged 60 years and above) US adults. Cognitive function was assessed as a composite score and on a test-by-test basis based on the Consortium to Establish a Registry for Alzheimer’s Disease Word List Learning Test, the Word List Recall Test, and Intrusion Word Count Test, the Animal Fluency Test, and the Digit Symbol Substitution Test (DSST). Depression was defined as clinically significant depression symptoms based on the standard cut-off point of the Patient Health Questionnaire-9 (PHQ-9) score of 10 or greater. Adjusted-logistic regression analysis was employed using survey weights to examine the former relationships. Sociodemographic factors, in addition to medical history and status in terms of self-reported chronic illness and the incidence of stroke or memory–cognitive function loss, were considered as covariates. Among 1622 participants of a survey-weighted 860,400 US older adults, cognitive performance was associated with clinically significant depression symptoms (*p* = 0.003) after adjustment. Most prominently, older adults with significant cognitive deficits had approximately two and a half times (OR: 2.457 [1.219–4.953]) higher odds for a PHQ-9 score above threshold compared to those with the highest performance. Particularly, those with lowest DSST score had increased odds of almost four times (OR: 3.824 [1.069–13.678]). Efforts to decipher the underlying aetiology of these negative disparities may help create opportunities and interventions that could alleviate the risks from depression, cognitive impairment, and associated consequences in older adults at a population level.

## 1. Introduction

Cognitive decline occurs as people age and poses significant repercussions on their ability to conduct daily activities and lead a quality life [1]. Late-life depression has been proposed to be an expression of cognitive impairment. A growing body of literature has highlighted a strong association between depression and cognitive impairments during adulthood, including deficits in word–list memory recall, working memory, and executive functioning [2,3,4,5,6]. Late-life depression has been linked to a more rapid decline in cognition, with older adults who report higher levels of depression performing worse on executive function tasks that require a greater mental workload compared to other cognitive domains [4,5]. The relationship between depression and cognition is complex, with ongoing debate as to whether bidirectionality exists [7]. Although it cannot be excluded, the majority of studies, particularly those with older populations, have consistently found that higher levels of depressive symptoms are a key risk factor for cognitive deficits and may serve as a precursor to cognitive impairment. To address the potential variations in and bidirectionality of cognitive function and late-life depression at a population level, we sought to examine the association between cognitive performance and clinically significant depression symptoms in older adults (aged 60 and above) in the US.

## 2. Methods

We extracted publicly available data from participants aged ≥60 years from the 2011 to 2014 survey cycles published in the National Health and Nutrition Examination Survey (NHANES).

Cognitive function was evaluated as a composite measure and a test-by-test basis based on the Consortium to Establish a Registry for Alzheimer’s Disease (CERAD) Word List Learning Test (WLLT), the Word List Recall Test (WLRT), and Intrusion Word Count Test (WLLT-IC and WLRT-IC), the Animal Fluency Test (AFT), and the Digit Symbol Substitution Test (DSST). The CERAD WLLT, WLLT-IC, WLRT, and WLRT-IC assess the immediate and delayed learning ability for novel verbal information and comprises three learning trials followed by a delayed recall challenge with a score ranging from 0 to 10. The AFT examines executive function by measuring categorical verbal fluency ranging from 3 to 39. The DSST consists of a performance challenge from the Wechsler Adult Intelligence Scale-III, which evaluates processing speed, sustained attention, and working memory, varying between 0 and 105. Higher test scores represent better cognitive performance.

Depression was measured using the Patient Health Questionnaire-9 (PHQ-9), a nine-item survey designed to diagnose the presence and frequency of depression symptoms over the previous 2 weeks. The score for each question ranges between 0 and 3 (0 = “not at all”, 1 = “several days”, 2 = “more than half the days”, 3 = “nearly every day”), reaching a total of up to 27. Clinically significant depression symptoms were defined using the standard cut-off point of the PHQ-9 score of 10 or greater.

Age, sex, ethnicity (race), family income status and size, education level, marital status, and US citizenship were treated as sociodemographic covariates. Medical history and status in terms of chronic illnesses based on the self-reported incidence of high blood pressure, diabetes, congestive heart failure, coronary heart disease, heart attack, stroke, memory–cognitive function loss, or cancer were also considered. Age was classified into groups between 60–69, 70–79 and ≥80 years of age. Ethnic groups comprised of Mexican American, other Hispanic, non-Hispanic White, non-Hispanic Black, non-Hispanic Asian, and other (multi) race. Family income status was categorised based on the family to income poverty ration (FIPR) as low–middle (FIPR < 1) and middle–high (FIPR ≥ 1). Family size of 6 or more individuals was defined as large and below 6 as small–medium. Education level was regarded as a college degree at minimum or below. US citizenship was assessed based on citizenship by birth or naturalization. Marital status comprised of four categories: never married, married, widowed/divorced/separated, and living with a partner. All covariates were treated as categorical variables.

Analyses were performed using survey weights to control for the multistage probability sampling design of NHANES. Participants without a response for any of the tests or questionnaires were excluded. A logistic regression model was used to assess and measure the association between cognitive performance and depression symptoms in older US adults after adjustment for all covariates. A sensitivity analysis treating cognitive performance and depression symptoms as continuous factors was also undertaken and the former relationship was reassessed with a linear regression model. The odds ratios (ORs) with 95% confidence intervals (CIs), and regression coefficients (β) were reported from the logistic and linear regression models. Statistical significance was established as *p* < 0.05. Statistical analysis was performed using the IBM SPSS statistics software (Version 28.0, IBM Corp., Armonk, NY, USA).

## 3. Results

Our study included 1622 participants (mean (SEM) age, 70 (0.2) years; 873 female participants (54%); 749 male participants (46%)) which represented a survey-weighted 860,400 US older adults, of whom 48% were non-Hispanic White (783) and 28% non-Hispanic Black (349). The majority of the participants held US citizenship (95% (1548)) and had a family income above the poverty threshold (82% (1335)). Most participants were married (54% (879)), of small family size (95% (1546)) and had a high school qualification or below at minimum (80% (1296)). A history of at least one chronic condition was reported in 91% of participants (1478), memory–cognitive function loss in 15% (248) and stroke in 9% (148). The average score for the CERAD WLLT was 18.8 (±0.1) out of 30, 5.8 (±0.1) of out 10 for the CERAD WLRT, 0.5 (±0.03) out of 12 for the CERAD WLLT-IC, 0.3 (±0.02) out of 10 for the CERAD WLRT-IC, 16.2 (±0.1) out of 40 for the AFT, 44.4 (±10.4) out of 100 for the DSST and 86.0 (±0.6) for their composite. Most participants (90% (1458)) had a score below the threshold for clinically significant depression symptoms. All baseline participant characteristics are described in Table 1.

**Figure 1:**
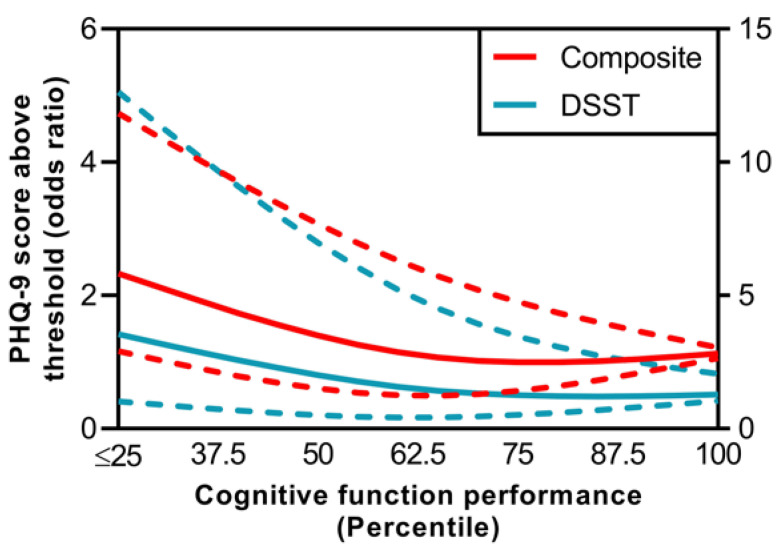
Logistic relationship between cognitive function and depression in older (≥60) US adults after adjustment for sociodemographic factors and medical history in terms of self-reported chronic condition and incidence of stroke or memory–cognitive function loss. Cognitive function was expressed as a composite score and on a test-by-test basis based on the Consortium to Establish a Registry for Alzheimer’s Disease Word List Learning Test, the Word List Recall Test, the Intrusion Word Count Test, the Animal Fluency Test, and the Digit Symbol Substitution Test. Depression was defined as clinically significant depression symptoms based on the standard cut-off point of Patient Health Questionnaire-9 (PHQ-9) score of 10 or greater. Significant associations between cognitive function including its components and depression symptoms are shown. Solid lines represent the estimated odds ratio and the area bound by the dashed lines represents the 95% confidence interval. Cubic splines with three knots were used for modelling. The highest quartile of cognitive performance and PHQ-9 score above threshold were used as the reference.

Cognitive function performance was significantly and negatively associated with clinically significant depression symptoms (*p* = 0.03) in older US adults after controlling for multistage sampling, sociodemographic and medical history covariates (Figure 1). In particular, participants with significant deficits (Q1; below the 25th percentile) and above average (Q3; above the 50th percentile) cognitive performance had approximately 150% (OR: 2.457 [1.219–4.953]) and 40% (OR: 1.378 [0.741–2.559]) increased odds of reporting a PHQ-9 score above threshold, when compared to those with highest cognitive performance (Q4; above the 75th percentile). Participants with cognitive performance below average (Q2; between the 25th and 50th percentiles) had smaller difference in the likelihood (OR: 1.016 [0.427–2.414]). An overall similar association between cognitive function and depression symptoms (*p* = 0.00028, β: −0.03) was observed in the sensitivity analysis.

On a test-by-test basis, cognitive performance in terms of DSST was significantly associated with depression symptoms (*p* = 0.006) in older US adults. Specifically, participants with cognitive performance in Q1 and Q2 had increased odds of almost 300% (OR: 3.824 [1.069–13.678]) and 200% (OR: 2.087 [0.659–6.609]) for PHQ-9 score above threshold when compared to those in Q4. Participants in Q2 had less variation in the likelihood (OR: 1.172 [0.360–3.816]) of reporting depression symptoms. Similar association between DSST and depression (*p* < 0.001, β: −0.044) was seen in the sensitivity analysis. No associations were observed for WLLT (*p* = 0.311) and WLRT (*p* = 0.838). Analysis based on WLLT-IC, WLLR-IC and AFT were not possible due to incomplete representation among quartiles.

## 4. Discussion

Using a nationally representative sample from NHANES 2011 to 2014, our cross-sectional study showed that cognitive performance including DSST was negatively associated with clinically significant depression symptoms in older US adults after adjusting for sampling, sociodemographic and medical history indicators. In particular, individuals with significant cognitive deficits had two and half times higher odds and up to four times compared to those with highest cognitive performance.

Our results align with previous research that examined the relationship between cognitive function and depression. Numerous population-based studies have shed light on the intricate relationship between late-life depression and its symptoms with cognitive decline [4,5,8,9,10], mild cognitive impairment [11,12,13] and dementia [13,14,15,16]. In fact, the links between depression and poor performance in cognitive function have led to the belief that it may act as an early indicator of dementia. In support of this, several studies have reported the association between depressive symptoms in late-life and decreased performance on specific aspects of cognitive function [5,10,17,18,19,20,21]. 

The trend of negative associations between late-life depression and cognitive function is evident, however some discrepancies exist. While some studies report associations across all domains of cognitive function, others only report in specific aspects and some only finding connections during certain comorbidity periods. In the same way, the rate of cognitive decline among older adults also appears at variance [5,22]. Likely, this may be largely due to the varied methods used in defining late-life depression and depressive symptoms, as well as the different means of testing cognitive function among older adults. Nevertheless, these studies along with ours contribute to a comprehensive picture of the impact of late-life depression on cognitive function, while the existing variations highlight the need for further research in this field.

The underlying causes of normal aging-related cognitive impairment have been widely discussed and investigated, with multiple mechanisms being proposed. At the cellular level, it is believed that the aging process contributes to decreased neuronal plasticity, partly by suppressing prefrontal cortex activation while reducing hippocampal neurogenesis [23,24]. These regions are particularly vulnerable to age-related changes in neural systems [25] and their attenuated activation could have a negative impact on cognitive function [26]. Likewise, a decline in brain volume, especially of the prefrontal cortex, has been linked to impairments in the executive cognitive function [27]. On the molecular level, a reduction in neurotrophic factors such BDNF [28] and neurotransmitters such as dopamine [29], combined with increased levels of glucocorticoids including cortisol [30], may lead to neurostructural and neurofunctional deterioration during aging, which can then result in diminished cognitive functioning.

The relationship between depression and cognitive impairment is influenced by various neurobiological factors often described by genetic, neuroimaging, and neurotrophin alterations [31]. For example, the epsilon 4 allele of the apolipoprotein E epsilon 4 gene (APOE-e4) is a known genetic risk factor for Alzheimer’s disease (AD) and has been linked to depression, with studies suggesting an additive risk for cognitive impairment and a synergistic effect in increasing the incidence of dementia and mild cognitive impairment [32,33,34,35,36,37,38]. Additionally, dysregulation of the hypothalamic-pituitary-adrenal axis in individuals with depression can result in elevated cortisol levels and consequent hippocampal atrophy, which is also seen in AD [39,40,41]. Depression and cognitive impairment have also been associated with changes in white matter, such as increased atrophy in regions affected by AD and reduced activation in neural networks [42,43,44,45,46,47]. Furthermore, brain-derived neurotrophic factor (BDNF) and transforming growth factor-beta1 (TGF-β1) are implicated in the pathophysiology of depression and cognitive impairment, with the BDNF Val66Met allele linked to late-life depression and the BDNF 11757C allele associated with depression in AD, and the C/C phenotype of TGF-β1 linked to an increased risk of AD and depression in AD patients [48,49,50,51,52,53].

The connection between cognitive function and depression likely also represents the drastic changes in lifestyles and circumstances that these critical age groups experience, as individuals reach retirement age [54]. Previous research has provided evidence that retirement is associated with a higher risk of depression and a modest but negative effect on cognition [55,56,57]. Most prominently, the decline in cognitive abilities from reaching retirement age can result in disruptions in social networks and withdrawal from social activities [58,59,60,61]. This can lead to symptoms such as a loss of interest, changes in appetite, psychomotor retardation, and sleep disorders, which further complicates changes in bodily and mental functions that come with aging [62]. In this way, retired older adults become more vulnerable to loneliness, negative emotions and social isolation in their daily lives with significant consequences on overall health and well-being.

All in all, the inverse relationship between cognitive function and depression among older adults underscores the significance of proactive diagnostic evaluations and social support that may help counteract the negative impact of cognitive decline on the onset of late-life depression. Most prominently, it highlights the critical requirement for environments and healthcare interventions that address the unique needs and challenges of this age group.

## 5. Conclusions

The interplay between late-life cognitive impairment and depression may in part appear correctable. Our moderate findings reinforce the need for health policy makers to design adaptable health practices that could potentially alleviate the risks from depression, cognitive impairment, and the associated consequences. Efforts to decipher the mechanism from which this inverse association emerges will maximise the effectiveness of health initiatives and improve public health benefits for older US adults.

## Figures and Tables

**Table 1:** Socio-demographic and medical history characteristics of participants (*n* = 1622). Values are expressed as count (percentage) unless otherwise specified.

Characteristics	Composition
**Age (year groups)**	
60–69	829 (51.1)
70–79	497 (30.6)
≥80	296 (18.2)
**Sex**	
Male	749 (46.2)
Female	873 (53.8)
**Ethnicity**	
Mexican American	123 (7.6)
Other Hispanic	137 (8.4)
Non-Hispanic White	783 (48.3)
Non-Hispanic Black	449 (27.7)
Non-Hispanic Asian	106 (6.5)
Other Race-including multiracial	24 (1.5)
**Family Income**	
Low–Middle	287 (17.7)
Middle–High	1335 (82.3)
**Family Size**	
Small	1546 (95.3)
Large	76 (4.7)
**Educational level**	
High School graduate or below	1296 (79.9)
College degree or above	326 (20.1)
**Marital Status**	
Married	879 (54.2)
Widowed	356 (21.9)
Divorced	214 (13.2)
Separated	50 (3.1)
Never married	91 (5.6)
Living with partner	32 (2.0)
**Citizenship**	
US Citizen	1548 (95.4)
Non-US Citizen	74 (4.6)
**Chronic Condition**	
Yes	144 (8.9)
No	1478 (91.1)
**Stroke**	
Yes	248 (15.3)
No	1374 (84.7)
**Memory–Cognitive function loss**	
Yes	148 (9.1)
No	1474 (90.9)
**CERAD WLLT (score)**	
Minimum	0
Average *	18.8 (0.1)
Maximum	30
**CERAD WLRT (score)**	
Minimum	0
Average *	5.8 (0.1)
Maximum	10
**CERAD WLLT-IC (score)**	
Minimum	0
Average *	0.5 (0.02)
Maximum	15
**CERAD WLRT-IC (score)**	
Minimum	0
Average *	0.3 (0.02)
Maximum	8
**AFT (score)**	
Minimum	3
Average *	16.2 (0.1)
Maximum	39
**DSST (score)**	
Minimum	0
Average *	44.4 (0.4)
Maximum	105
**Composite Cognition (Score)**	
Minimum	15
Average *	86.0 (0.6)
Maximum	165
**PHQ-9 thresold (Score)**	
Below 10	1458 (89.9)
10 or above	164 (10.1)

* Values expressed as mean (standard error). AFT = Animal Fluency Test; CERAD = Consortium to Establish a Registry for Alzheimer’s Disease; DSST = Digit Symbol Substitution Test; PHQ-9 = Patient Health Questionnaire-9; WLLT = Word List Learning Test; WLRT = Word List Recall Test; WLLT-IC = Word List Learning Test–Intrusion Word Count; WLRT-IC = Word List Recall Test–Intrusion Word Count.

## Data Availability

The data sets analysed in this study can be accessed in the 2011–2014 National Health and Nutrition Examination Survey (NHANES; https://www.cdc.gov/nchs/nhanes/index.htm, accessed on 23 December 2022).
